# Explanations of the political gridlock behind international circular economy: Waste Ban narratives in the China-EU cooperation

**DOI:** 10.1007/s13280-022-01758-4

**Published:** 2022-07-19

**Authors:** Anran Luo, Fabricio Rodríguez, Sina Leipold

**Affiliations:** 1 Chair of Societal Transition and Circular Economy, University of Freiburg, Tennenbacher Str. 4, 79106 Freiburg, Germany; 2 Department of Environmental Politics, Helmholtz Centre for Environmental Research - UFZ, Permoserstr. 15, 04318 Leipzig, Germany; 3 Chair of Environmental Politics, University of Jena, Bachstr. 18k, 07743 Jena, Germany; 4 Institute of Sociology, Friedrich Schiller University Jena, Bachstraße 18k, 07743 Jena, Germany; 5 Arnold Berstraesser Institute (ABI), University of Freiburg, Windausstr. 16, 79110 Freiburg, Germany

**Keywords:** China, Circular economy, Development, European Union, Waste Ban, Waste trade

## Abstract

China and the European Union (EU) signed the first international circular economy (CE) agreement shortly after China issued the ‘Waste Ban’ (WB) on the import of 24 categories of solid waste. While the WB gained global attention, limited research addresses its political implications for international CE. Based on 72 expert interviews, 52 documents and participant observation, we study political WB narratives and corresponding perceptions of ‘Chinese’ and ‘European’ agency in the China-EU case. Our results show a political gridlock in China-EU CE coordination: the main narratives on the WB diverge on the very roles and rules of CE cooperation, scales of implementation, and the assessment criteria for environmentally and socially sustainable CE(s). To enable fruitful CE cooperation, we suggest three critical points for reflection and negotiation: (1) overcome ‘student–teacher’ roles rooted in linear development that run counter to CE ideas; (2) reconsider the focus on international and national waste diversion, instead of local waste prevention, that propels global waste markets sustained by increasing waste volumes; and (3) evaluate both the benefits and impediments of (new) trade relations for a CE cooperation that contributes to environmental and social sustainability.

**Supplementary Information:**

The online version contains supplementary material available at 10.1007/s13280-022-01758-4.

## Introduction

China’s 2018 Waste Import Ban (WB), which (re)cast the global waste trade in the global environmental spotlight, has become an emergent focus in circular economy (CE) debates (The Economist 2018; Qu et al. 2019; Pacini and Yeoh 2021). A prominent international narrative for addressing pressing environmental problems of extraction, resource use and waste management, CE aims to turn ‘waste’ from a problem into a solution (Mckinsey Center for Business and Environment 2016). The global waste trade, which turns ‘unwanted waste’ into ‘resources’ has thus become a CE practice (Gregson et al. 2015; Romero-Hernández and Romero 2018; O'Neill 2019). The WB interrupts the global waste trade by stopping China’s import of 24 kinds of solid wastes, including plastic, paper, and other types of low-grade scrap (World Trade Organization 2017). Yet, literature also suggests the WB to be a positive force for international CE and environmental impact mitigation (Wen et al. 2021; Yoshida 2022).

To make sense of this discrepancy, we discursively analyze the relationship between the WB and CE in the context of the first international CE cooperation, using the Memorandum of Understanding on Circular Economy (MoU) between China and the EU as an entry point (European Commission and Chinese Development and Reform Commission 2018).

In the years prior to the WB, China was the single largest waste importer while the European Union (EU) or EU-28 was collectively the largest waste exporter (Velis 2014; Brooks et al. 2018). To give an indication of the WB’s effect in trade volumes between the two major economic actors, the EU exported about 1.4 million tonnes of plastic waste to China in 2016. However, in 2018, its exports of plastic waste to China reduced significantly to 50 thousand tonnes (Eurostat 2020). For detailed analyses of the WB’s implications from perspectives of the global waste trade or material flows, see Brooks et al. (2018), Tan et al. (2018), Wang et al. (2019), and Qu et al. (2019).

In addition to the WB’s quantitative magnitude for China and the EU’s waste trade, the China-EU CE cooperation presents an opportune discursive case for studying the WB because the MoU focuses on sustainable resource use and waste management as key fields of cooperation (European Commission and Chinese Development and Reform Commission 2018). Our study therefore contributes to the literature by providing an in-depth account of the systems of meaning and signification underpinning the WB and its consequences for a global CE. We ask the following research questions:

1. How do stakeholders perceive the WB in relation to the China-EU CE cooperation and how do they react to it discursively?
2. What do WB narratives tell us about perceptions of international CE and;
3. What are their implications for international CE?

The results of this analysis provide critical knowledge not only for CE scholars, but more importantly, for scholars interested in identifying suitable and politically feasible socio-economic frameworks to address the growing challenges posed by global environmental change. Our study on narratives and perceptions of the WB in China-EU CE cooperation serves as a rare opportunity to answer calls (e.g. McDowall et al. 2017) that we need more insights into how these two CE frontrunners conceptualize a global CE as well as wider global environmental challenges of waste.

## Discursive agency approach

Terminology is important for discussing the WB. Following O'Neill (2019), we use the term ‘waste’ to refer to non-reusable materials and the term ‘scrap’ to refer to reusable materials for easy comprehension. Furthermore, we acknowledge that ‘developed’ and ‘developing’ are contested categories for describing nations because they obscure the relations of unequal exchange that sustain and reinforce these hierarchies. Yet, we refer to this terminology in the way that the interviewed stakeholders themselves use it to make sense of China-EU cooperation in global waste management. These terms are also used without critical reflection in different policy documents we look at in this article.

To analyze how the WB affected China-EU relations in the global waste regime and what this means for global CE aspirations, this paper draws upon the discursive tradition of interpretive policy analysis, which has gained prominence in global environmental politics since the 1990s (Litfin 1994; Hajer 1995; Bäckstrand and Lövbrand 2006). Our analysis focuses on the way national (Chinese) and supranational (European) discourses and agencies are constructed in relation to the WB, and what the reconfiguring dynamics and qualities of this relationship mean for the conceptualization and potential operationalization of a global circular economy. We believe that much can be learned from this regarding the political prospects of a global CE because discourses and narratives illuminate the underlying meaning structures that foster, and/or hinder, the coordination of actions between actors lacking common policy frameworks such as China and the EU (Sharp and Richardson 2001; Dryzek 2013). These meaning structures are critical for explaining current policy processes and anticipating how they might develop in the future because they determine how social actors convert human difficulties into policy problems, constitute policy instruments, and create coalitions of support or opposition (Fischer and Forester 1993; Roe 1994; Yanow 2000; Fischer and Miller 2017).

Specifically, we employ the Discursive Agency Approach (DAA) (Leipold and Winkel 2017), which employs the analytical elements of political institutions and discourses from Argumentative Discourse Analysis (ADA) (Hajer 1995) and adds its own elements of discursive agency as heuristics for our analysis. This means we consider global policy making to be a continuous struggle over establishing political truths and corresponding policies and institutions, which takes form through policy discourses (Leipold and Winkel 2017). We conceive of such discourses as a sum of (topically related) communicative interactions between social actors (Keller 2013) and the definition of “narratives” (or “storylines”) as a subset of overarching discourses (Hajer 1995). ‘Narrative’ is defined as a story ascribing meaning to social or physical phenomena by connecting a sequence of events and actions in a plot, including, excluding, and emphasizing problems, actors, and events and, thus providing an interpretation of who or what is significant and when (Kaplan 1993; Hajer 1995; Feldman et al. 2004). We derived analytical categories from this definition to operationalize our narrative analysis, which is made explicit in the results, and visualized in Tables 2, 3, and 4 in the results section. Following Hajer (1995), we consider dominance of a narrative to be constituted by discourse structuration, where actors draw on the ideas, concepts, and categories of a given discourse to maintain credibility. Discourse institutionalization is defined as when a given discourse is translated into institutional arrangements.

We adapt Leipold and Winkel (2017)’s definition of ‘discursive agency’ for the international context where there are more diverse groups of actors: discursive agency is “an actor’s ability to make him/herself/themselves as well as other actor(s) a relevant agent in a particular discourse by constantly making choices about whether, where, when, and how to identify with a particular subject position in specific story lines within this discourse” (p. 15). The adaptation emphasizes Leipold and Winkel (2017)’s own conceptualization that the degree of collectivity of an actor, whether as individuals, collectives, or coalitions of individuals or collectives, to be a positional characteristic dependent on the perceptions of actors in the discursive policy arena. The DAA assumes a dialectically constituted agency. On the one hand, discursive structures produce the preconditions for agency by influencing not only what stakeholders think or do but also who they are as political subjects. On the other hand, it is the actors who (re)produce and thus do discourse, shaping institutions and transformational pathways (such as CE) in a particular direction.

**Fig. 1:**
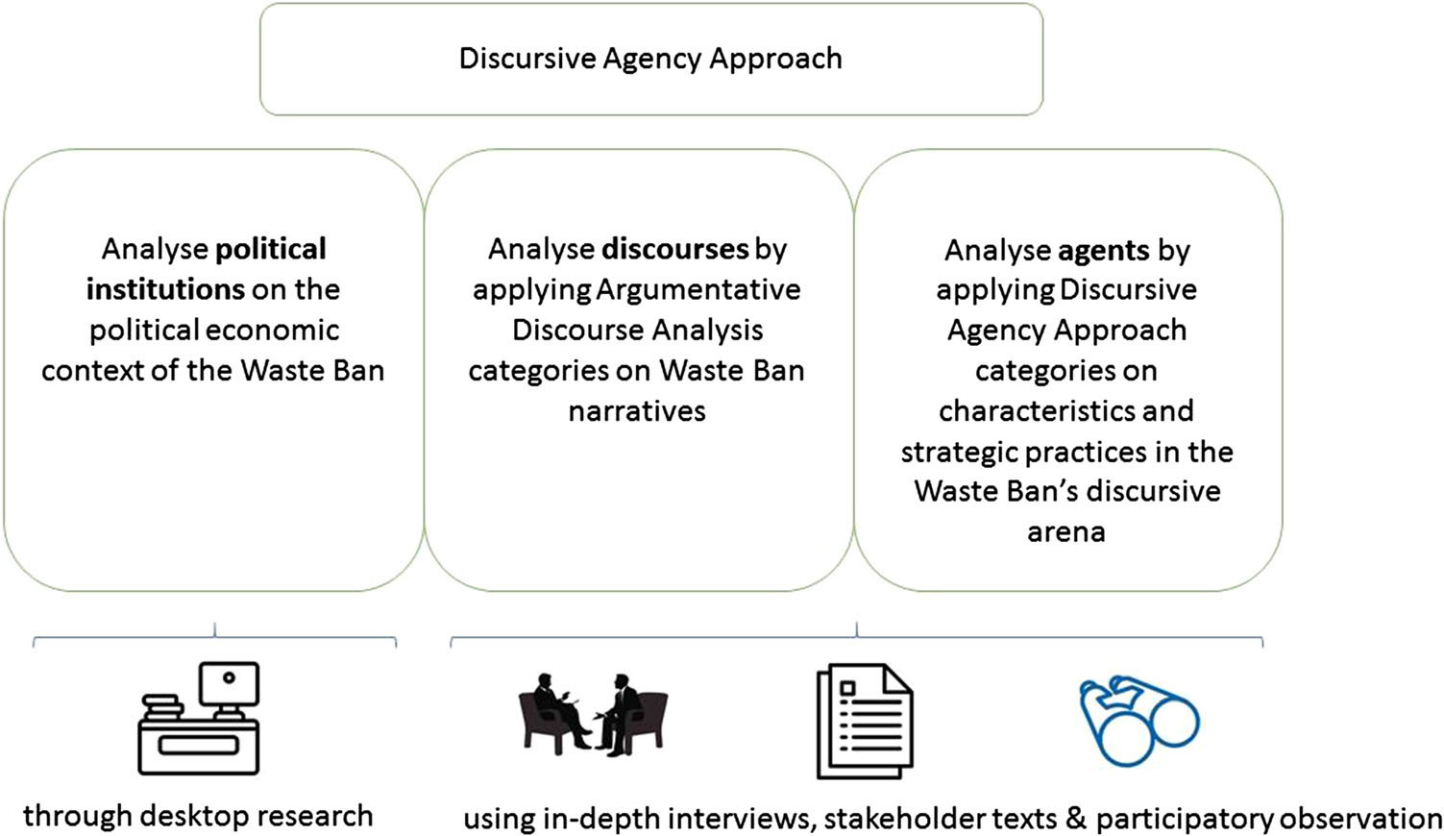
Analytical approach and methods

By using DAA, we focus on the ways discursive practices, expressions of intersubjective relations, produce particular kinds of narratives and agencies, truth claims and corresponding policies and institutions. They, thus, shed light on how stakeholders conceptualize and shape an international policy field, their own and others’ discursive agencies within it, and its future development. Figure 1 outlines how we map and analyze the WB institutions, discourses and agents in the context of the China-EU CE cooperation:

## Materials and methods

To map and analyze the WB institutions, discourses and agents in the context of the China-EU CE cooperation, we collected data from key stakeholders who work in relevant fields close to the WB, contribute to China-EU CE discourse or who have worked on China-EU projects related to waste management and circular economy. The data set is comprised of:

- 20 explorative interviews that helped to map the stakeholder field
- 49 semi-structured interviews with a focus on the WB (between 30 and 120 min in length, recorded and transcribed)
- 23 semi-structured interviews with a broader focus on China-EU CE (between 30 and 120 min in length, of which 12 were recorded and transcribed; 11 could not be recorded because interviewees did not give consent, these have been documented using on-site notes as well as follow-up memory protocol)
- 12 documents related to WB (e.g. WTO notifications and filings, Basel Convention documents, Chinese official documents, press releases, media articles, trade association documents)
- 40 documents related to China-EU CE (e.g. MoU, environmental dialogues, joint declarations and event programs, press releases, speeches, media articles, publications)
- Participant observation at the International Circular Economy Conference and Exhibition in Beijing (November 2017), 2019 Circular Economy Stakeholder Conference in Brussels (March 2019), and the World Circular Economy Forum in Helsinki (June 2019)

The data was gathered between October 2017 and August 2019. In a first step, interview guides were drafted based on our research questions and DAA and ADA’s analytical elements (Hajer 1995; Leipold and Winkel 2017). Explorative interviews were conducted in autumn 2017 and early 2019 with experts knowledgeable on different aspects of the field or with an overview of the topic but who were not directly involved. These interviews provided important background and context information for the WB and CE in China and the EU, guidance for setting our case boundaries as well as insights for the formulation of the interview questionnaires. Next, we gathered relevant communication and policy documents through desk research, which together with information gathered from the helicopter interviews, suggested potentially relevant interviewees. Finally, the in-depth interviews were conducted between January and August 2019. Based on the initial search, a list of 50 individuals or organizations was compiled. The individuals or organizations were then contacted and a set of five interviews was conducted. The interview list was refined and, where necessary, expanded using a snowballing method according to information gathered in the initial interviews. This process was repeated until the remaining individuals could not be reached for an interview (after five attempts) or refused the interview. In the end, 72 interviews could be secured in English and Mandarin Chinese and were transcribed according to the recordings without translation.

The interview data was analyzed deductively, based on categories deduced from DAA and ADA as well as from our interview guide, and inductively, inspired by grounded-theory techniques using the coding software MAXQDA (Saldaña 2015). Further documents and participant observation data from relevant stakeholder events were analyzed to contextualize and complement the interview results. In the results section, direct quotations from Mandarin Chinese interviews are translated into English for comprehension purposes.

To assure the protection of interviewees’ personal data, aggregated stakeholder categories (e.g. A = academic institutions) have been developed for the purpose of referencing interviews in this article (see Table S1 in Supplementary Information). The interviews in each category were numbered according to the interview date (e.g. A1 = first interviewee from this category, P7 = seventh interviewee from this category). The codes do not represent the order of interviewees’ affiliations presented in Table S1 in Supplementary Information).

## Results

All results are based on the interviews, documents and participant observation and complemented by a targeted literature search. The documents and interviews helped to identify the most important groups of agents, who are stakeholders characterized as particularly relevant in the discursive struggle that the WB launched.

We identify ‘China’ and ‘the EU’ as key collective discursive agents not because we view them as unitary actors but because interviewees often used this shorthand to refer to the official agents of the respective country/supranational organization. Chinese agents tend to use the phrase “we, China” (referring to Chinese people or the nation and not any particular agent) when speaking about the self. For example, a recycling industry official when seeking to differentiate her own view from high-level official perspectives, said “We, China, still need to develop. We still need plastic” (CH_I4). In this way, ‘China’ was also sometimes used to signify the waste-importing ‘industrializing’ countries. Most agents referred to the European Commission when using the shorthand ‘the EU’; however, many EU recycling industry agents referred to the European continent as their associations represented recyclers across the continent. Chinese agents sometimes conflated ‘the EU’ with other waste-exporting industrialized nations such as the US, or referred to it in association with western nations. For other details of diversity in agents, please refer to Table 1.

**Table 1:** Key discursive agents relevant for the Waste Ban in relation to China-EU CE cooperation

Key collective discursive agents:	‘China’: shorthand for the Chinese nation, ‘developing’/ ‘industrializing’ countries, & official Chinese agents	‘EU’: shorthand for the European continent, western nations, ‘developed’/ ‘industrialized’ nations, & official European agents	International organizations
Diverse discursive agents identifying with/ identified as key collective agents:	Government agents: National Development & Reform Commission, State Council, Ministry of Ecology and Environment Chinese CE scientists working at government and academic institutions: agents with policy advisory roles Agents representing individual recyclers: active in international CE community but with limited policy lobby in China	Agents representing the European Commission, DG Environment, DG Grow Agents representing EU member states considered more advanced with CE such as the Netherlands, Germany, France Agents representing individual recyclers and packaging companies in the EU: lobby groups with influence at the Commission level	International (environmental & economic) agents acting as intermediaries but also sites of discussion for ‘China’ & the ‘EU’, e.g. OECD, WTO, Greenpeace, EMF

In the next section, we use DAA’s analytical elements: institutions, discourses, agents and practices to take stock of stakeholder perceptions of the global waste trade before the WB. Thereafter, we do the same for stakeholder perceptions after the WB.

### The past: the evolution of the global waste trade

**Fig. 2:**
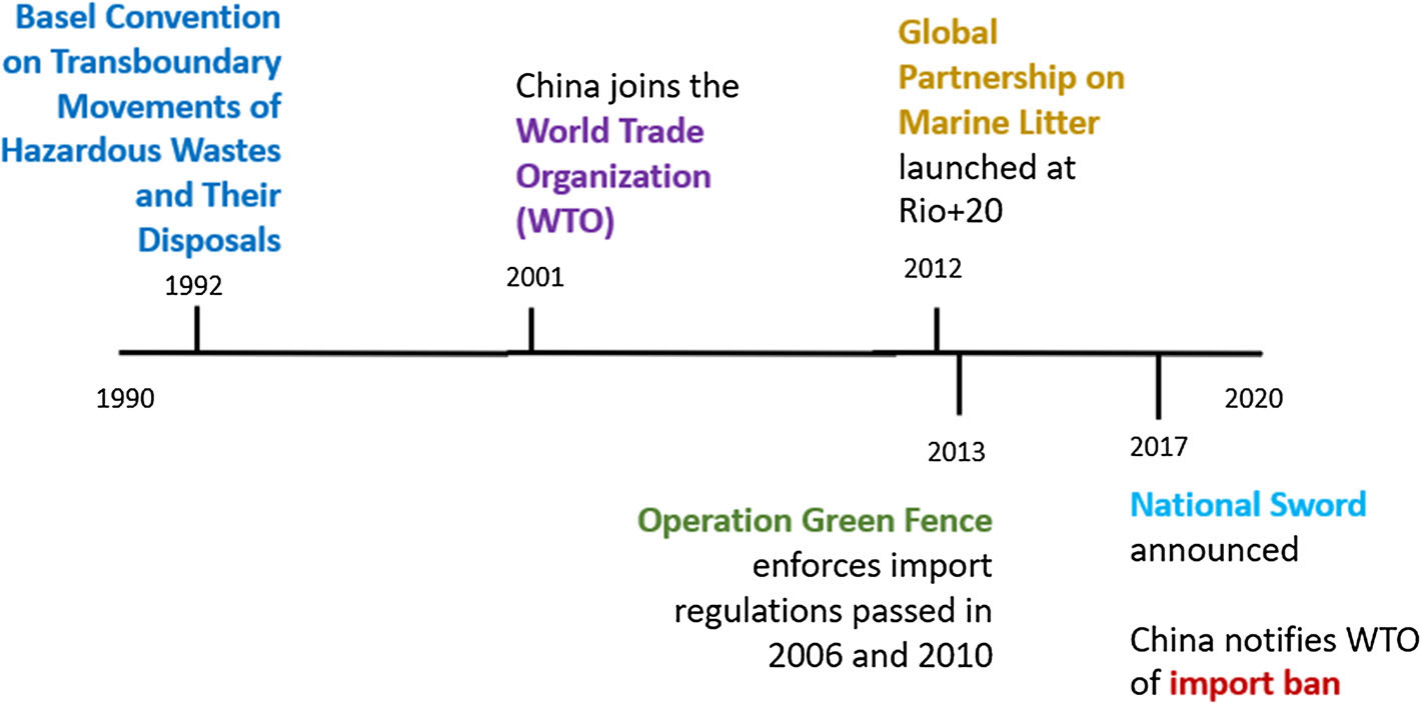
Political institutions in the evolution of the global waste trade

The origins of the global waste trade can be traced back to the 1980s. Trade volumes skyrocketed between the 1990s and 2010s. In this time period, China was the single largest waste importer while the EU-28 was collectively the largest waste exporter (e.g. CH_R3; see also Velis 2014). The World Trade Organization (WTO; and its predecessor the GATT) and the Basel Convention on the Control of Transboundary Movements of Hazardous Wastes and Their Disposal (Basel Convention) regulated and facilitated this global waste trade (e.g. EU_I11, CH_R9). China’s entrance to the WTO in 2001, and the increasing trade volumes between China and the rest of the world, paralleled the rapid growth of the global waste trade (e.g. CH_I4; see also O’Neill 2019). As the industrialized countries such as many in the EU imported increasingly more manufactured products from China, containers arriving at EU ports with goods and returning to China with waste scrap became common practice (e.g. IO_1, EU_I7, IO_2; see also O’Neill 2019) (Fig. 2).

The WTO’s institutional framework, intended to foster free trade between the world’s nations and ensure “a level playing field for all, thus contributing to economic growth and development” (World Trade Organization 2022) shaped the global waste trade discourse that contextualizes the WB’s discursive arena. This narrative is not contested as stakeholders agree that the waste trade was mutually beneficial for exporters and importers at the time, and thus importing and exporting countries such as China and the EU were both perceived to enjoy agencies as ‘winners’.

The waste trade paralleled China’s rapid manufacturing-based economic expansion and offered a solution to its problems of resource scarcity and low technology base. Importing ‘foreign’ waste was considered very valuable: the wastes contained high quality materials, especially plastics, which could be processed and upgraded through low-cost technologies and turned into high value inputs (CH_I5). Simultaneously, exporting wastes was a cost-effective solution for EU and other waste-exporting regions to manage waste-related environmental problems as consumption soared, in part, as some argue, due to cheaper products from China. The waste trade constituted a way of diverting from landfill without extra investment into alternative domestic waste management programs and “it also found a second life for unwanted materials” (IO_1).

As the WTO was a pivotal institution for the evolution of the global waste trade, the Basel Convention shapes its transition as the political struggles over the WB plays out. While the 1992 Basel Convention prohibits the transborder movement of “hazardous wastes” from “developed to less developed” countries (Basel Convention 1992), the attempt to come to a common definition on “hazardous waste” has been a challenge. Plastic is an example of a category of waste that has escaped such a definition. Prior to 2016, China primarily imported waste plastics (EU_I7; see also Velis 2014), which served as an important material input to build China’s state-led export-oriented economic growth model and to enhance its industrial modernization (CH_R1, CH_I5; see also Qu et al. 2019).

According to almost all stakeholder groups, the increasing visibility of the marine litter discourse on the global stage played a key role in shifting perceptions on plastic and by extension on the global waste trade. Over the last decades, the topic of marine litter has become a global topic. For example, in 2012, the United Nations launched a Global Partnership on Marine Litter at the Rio 20 + Conference (UNEP no date). More recently, the Ellen MacArthur Foundation famously coined the phrase: “There will be more plastic than fish in the sea by 2050” (World Economic Forum et al. 2016). Although the WB targets 24 kinds of solid wastes, plastic waste is the first on the ban list and by far the most discussed.

### The present: the global waste trade in transition and the Waste Ban

**Fig. 3:**
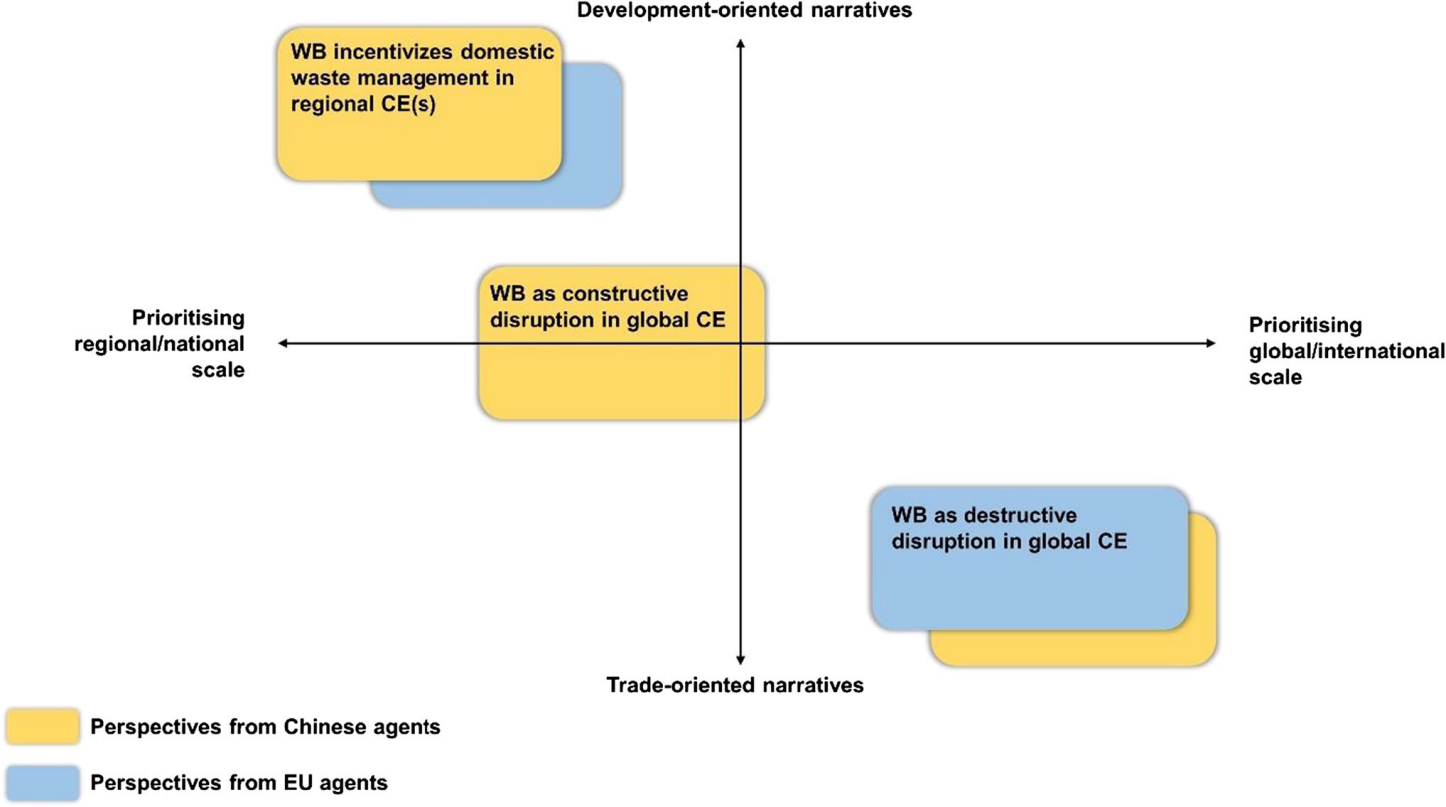
Waste Ban narratives mapped against development/trade orientation and scale prioritization

By issuing the WB at the WTO, China implemented a governance strategy (Leipold and Winkel 2017), which changed the rules of the global waste trade and forced all stakeholders into a transitional stage. In the context of the China-EU CE cooperation, which coincides with this period, Chinese and EU agents employed various discursive strategies to justify and rationalize their perceptions and reactions towards the WB. A key strategy is the creation of narratives to strengthen the reception of their perspectives. Our analysis identified three particularly prominent narratives (see Fig. 3) that can be mapped according to their prioritization of scale and orientation towards either trade or development. While the first narrative, *WB as constructive disruption in global CE*, features only perspectives from Chinese agents, the latter two narratives, *WB as destructive disruption in global CE* and *WB incentivizes domestic waste management in regional CE(s)*, draws on perspectives from Chinese and European agents.

#### WB as constructive disruption in global CE

The first narrative *WB as constructive disruption in global CE (*hereafter referred to as *‘WB as constructive disruption’*) is the most representative of the official Chinese perspective (Table 2). It structures the narratives of most Chinese agents, except for select recycling industry representatives. It is institutionalized in the WB itself, in China’s WTO responses to complaints against the WB, but also in two prior campaigns. Operation Green Fence (2013) and National Sword (2017), two Chinese customs operations, signaled changes on the horizon for the global waste trade (e.g. CH_P2, CH_R4; see also Resource Recycling 2018). While Green Fence focused on enforcing the quality of the imported waste, National Sword alternated between cracking down on criminal activity such as permit fraud and import quality (EU_I11, IO_2). The official objectives of both campaigns were to ensure compliance with earlier import regulations and amendments from 2005, 2008 and 2009: Catalogues of Imported Wastes Management according to the Law of the People’s Republic of China on Prevention and Control of Environmental Pollution Caused by Solid Waste and the Basel Convention (The General Administration of Quality Supervision, Inspection and Quarantine 2005; Chinese Environmental Protection Administration 2008; Ministry of Environmental Protection of the People’s Republic of China 2009). Although Green Fence and National Sword signaled changing winds to some waste industry stakeholders (EU_I4), many stakeholders in the EU paid little attention to them as they were considered domestic scale campaigns. It is China’s filing at the WTO to stop importing 24 kinds of solid wastes including plastic, paper, and other types of low-grade scrap (World Trade Organization 2017) that grabbed the world’s attention. For the official Chinese agents who created this narrative, the WB is the natural endpoint of a series of enforcement efforts to clean up the import of foreign wastes.

**Table 2:** Official development-oriented ‘China’ WB narrative

Narrative	*‘WB as Constructive Disruption’*
Problem	Pollution of air, water and soil
Cause	Poor behavior from waste exporters from ‘the EU’: criminal, smuggling, contamination
Consequence	China had to bear social and environmental cost of separating scrap from waste due to contamination, also illegal activity
Solution	WB stops entry of contaminated and hazardous wastes from entering China
Benefits	Inspires other importing countries to also issue WBs; opportunity for the export of Chinese recycling technologies

**Table 3:** Trade-oriented EU counter-narrative

Narrative	*‘WB as Destructive Disruption’*	
	Perspectives from Europe	Perspectives from China
Problem	Sudden implementation of WB by China	High-level Chinese political decision to implement WB
Cause	Chinese protectionism, authoritarianism and lack of transparency	International marine litter discourse damaged China’s environmental image
Consequence	Disruption to the global waste trade Countries without recycling capacity flooded with waste Recycling industry hurt in EU and China	Disruption to the global waste trade Countries without recycling capacity flooded with waste Recycling industry hurt in EU and China
Solution	Repeal the WB through WTO China-EU CE cooperation should redefine ‘waste’ and ‘scrap’ Close the loop on plastic	Repeal the WB through WTO China-EU CE cooperation should redefine ‘waste’ and ‘scrap’ Close the loop on plastic
Benefits	Re-enable global free trade of scrap	Re-enable global free trade of scrap

The core premise of *‘WB as constructive disruption’* is that the WB disrupts the entry of contaminated and hazardous wastes from entering China, inspires other waste-importing countries to follow suit, and spearheads a regime-change in the global waste trade. Agents reproducing this narrative argue that China has developed to the point where it is no longer so resource scarce that it would continue to tolerate illegal dumping activities and “pay the (environmental and economic) price” of waste processing from other countries to obtain scrap (e.g. CH_R6, CH_P5). Instead, after years of studying and learning from EU regulations (among other ‘developed’ nations e.g. Japan, US), China is now ready to be a rule-setter in the waste regime. Agents perceive the WB as a regulatory practice that raises China’s waste import standards against foreign exporters’ irresponsible practices. As one Chinese CE researcher emphasized: “…if the waste is pure it can be rather good, but when the foreign waste is mixed with toxic and hazardous substances…and even some illegal items, then it is bound to be prohibited it” (CH_R5). Chinese stakeholders from policy and industry in particular envision that China, through its Belt and Road initiative and cooperation mechanisms such as the EU-China CE MoU, will have the opportunity to export cost-effective waste management technology and knowledge to the EU. Other collaborations could include research into new (bio)materials, as well as partner with the EU to help other developing countries, for example in Africa, with environmental technology and policy development (CH_P5, CH_I2). Agents argue that now that China is further along its ‘development’ path, “the ‘cooperation point’ with western countries naturally shifts from ‘low-grade’ waste treatment to ‘high-grade’ technological innovation” (EU_P13, IO_5).

To create this narrative, agents employ various mutually reinforcing discursive strategies: ‘delegitimization’, ‘exclusion’, ‘divide and conquer’, and ‘coalition building’ (Leipold and Winkel 2017, p. 526). They delegitimize the EU’s, and other western nations’ environmental records by showing that they rely on externalizing hazardous and even illegal wastes to ‘developing’ nations and essentially perpetrating environmental crime. The delegitimization strategy puts the blame for the problem of environmental pollution in China squarely on the waste-exporting nations and excludes China’s own waste policy-implementation gap from the discussion. The narrative also simultaneously pits waste-exporting nations against waste-importing nations while building a coalition between China and waste-importing nations, where China is attributed developmental agency for leading the way with good waste management practices.

Last but not least, this narrative also ‘employs normative power’ as a discursive strategy by connecting the WB to the accepted value of ‘learning’ (Leipold and Winkel 2017, p. 526) and promotes ‘the EU’ and ‘China’ in a ‘teacher-student relationship’. We attribute this analogy to this strategy because it describes the myriad ‘inspiring’, ‘learning’, and ‘knowledge-sharing’ references stakeholders, especially official Chinese agents but also scientists, used when relating the two political agents. It primarily emphasizes the WB is a standard-setting practice that protects the environment, comparable to European regulatory regimes for chemicals (REACH: Registration, Evaluation, Authorisation and Restriction of Chemicals) and for electronic wastes (WEEE: Waste Electrical and Electronic Equipment Directive). It also suggests an emergent agency for China in the role of the teacher in realms such as cost-effective waste management technologies, which ‘the EU’ and other western countries have not invested in due to exporting waste in the last decades. This new agency further brings the *‘WB as constructive disruption’* narrative in the direction of a trade orientation as well as to the global scale, signaling that it is not completely stepping out from the WTO institutions. As various agents stressed, while China “closes off waste cooperations” with the world, it hopes to open “technology cooperations” through the Belt and Road Initiative (IO_5, CH_I2, CH_R3).

#### WB as destructive disruption in global CE

The second narrative (Table 3) *WB as destructive disruption in global CE (*hereafter referred to as ‘*WB as destructive disruption’*) is the most prominent counter-narrative produced against the *‘WB as constructive disruption’* narrative, primarily within the EU Commission, the EU recycling organizations, and echoed by Chinese recycling organizations and agents from trade-oriented international organizations. This narrative is institutionalized in the WTO filings against the WB, which means it also has support from other waste-exporting countries such as the US.

*‘WB as destructive disruption’* stresses that the EU’s supply of scrap remains important for China and that environmental problems of the global waste trade should be addressed but not through a hard instrument such as a ban which goes against global free-trade (IO_1). An EU Commission representative’s statement points out the precise problem for Europe: “China had a policy last year … on banning waste which we think it was a good move for- for them and we applaud them for doing it but we despise them for doing it in a very abrupt way….harming European companies” (EU_P7). A recycling industry representative sums up the narrative with regards to scrap with this analogy: “China has been throwing (out) the baby with the bathwater… I fully understand the need for China to protect its environment, I mean, it's absolutely crucial. Eh, but…the magnitude of, of the measures was kind of disproportionate” (EU_I11). According to this narrative, the sudden implementation of the WB resulted in major losses to the European waste management industry and caused waste to pile up in European ports instead of reentering the value chain. It also led to waste streams being shifted from China to Southeast Asia, where recycling capacity is still immature and technological capability is not yet best-in-practice, resulting in waste leakage and environmental pollution.

Two potential solutions are presented in this narrative. First, China-EU cooperation through bilateral and plurilateral talks, including but not limited to dialogues under the CE MoU, could result in joint re-definitions of trade rules governing what is ‘waste’ and what is ‘scrap’ to enable a more environmentally and socially sustainable free trade of secondary raw materials, as this generates global economic gains (eg. EU_I10, EU_I11, IO_1). Future cooperation with China should be co-funded and mutually beneficial as the EU no longer considers China as a developing country (European Commission 2019). A second solution is an effort to repeal the WB altogether through the WTO dispute resolution channel. The EU has filed complaints against the WB at the WTO together with other waste exporting nations with hopes to re-stabilize the WTO regulatory environment (eg. EU_I10, IO_8).

*‘WB as destructive disruption’* employs a discursive strategy of scientification to counter *‘WB as constructive disruption’*. It characterizes a general skepticism towards Chinese authoritarianism, intransparency as well as protectionism. The WB is considered an example of all three because of perceptions that it was implemented very quickly by Chinese authorities, without forewarning or consultation rounds with other stakeholders, and without reference to science. A representative of the European Commission summarized this view by arguing that China put the ban into place “without providing justifying evidence for their choices but putting artificial purity levels on recycling streams that they are not justified by research, but they smell more like protectionism…” (EU_P7).

A minority Chinese perspective in this narrative, represented primarily by agents from the recycling industry, frame the WB as a counter-narrative to the marine litter discourse led by international actors such as UNEP, EMF and the EU (e.g. Plastics Strategy) but also Chinese environmental NGOs and activists (the documentary Plastic China was cited to be particularly influential) that frames China as a perpetrator of marine litter. These voices agree with their European counterparts that the global waste trade still benefits Chinese development and should continue, but support the official China WB narrative in that the WB was not a sudden decision but rather a gradual response to environmental problems. However, they provide a more nuanced perspective by shedding light on how the perception of the severity and priority of the waste problem in China to be co-constructed between domestic and international environmental agents.

Like *‘WB as constructive disruption’*, ‘*WB as destructive disruption’* promotes the EU and China in a ‘teacher-student relationship’. However, here the analogy is used more as a delegitimating strategy. In terms of policy and technology, agents reproducing this narrative continue to see China as EU’s ‘student’, keen to learn what it can from Europe’s advanced economies. While China’s increasing agency is recognized, Europe is deemed to be so far ahead in ‘development’ that it is the natural environmental leader and standard setter. When referring to CE-related waste policy, a European Commission representative said: “We believe that we inspire- it's logical to inspire them because we are more developed and it's logical that they- they have to- um, they need to- we need to get inspired by others as well, huh?” (EU_P7). This quote is symbolic because it captures the changing dynamics within the ‘teacher-student’ relationship, where the relationship is still seen primarily as where the EU is the ‘teacher’ but where the knowledge balance is beginning to shift.

#### WB incentivizes domestic waste management in regional CE(s)

The third narrative (Table 4) *WB incentivizes domestic waste management in regional CEs* (hereafter referred to as ‘*WB incentivizes domestic waste management’*) is popular with all groups of stakeholders, including many international organizations and NGOs, and structures their rationales for the WB as promoting regionally responsible CEs. Official and unofficial Chinese agents, especially Chinese CE scientists are co-creators of this narrative. EU policy stakeholders at development agencies, EU member state governments, as well as the European Commission co-created this narrative on the European side. While this narrative is institutionalized with Chinese waste management policies such as Zero-Waste Cities and EU CE policies such as the Single-plastic directive, it stops short of materializing in any joint China-EU cooperation despite the MoU’s stated goal to cooperate on waste.

For China, *‘WB as domestic waste management incentive’* emphasizes that the WB cuts off foreign supply for the Chinese recycling industry, which incentivizes formal and centralized recycling systems and supports existing national waste management initiatives such as Zero-Waste cities (CH_P7, CH_NGO1). It also puts an end to informal systems of waste pickers, their precarious working conditions and the related negative images of China as the world’s waste dump (e.g. IO_5, IO_6). The WB is presented as a necessary measure and a solution in this narrative, accompanied by visions of a national CE, where waste recovery and waste-to-value processes occur within China’s borders through comprehensive waste management infrastructures and institutions for waste collection, sorting, recycling, and incineration. Building a comprehensive waste management system and focusing on China’s domestic waste streams is considered a step towards building an ‘ecological civilization’: China’s version of sustainable development (e.g. CH_P7, CH_NGO1, IO_9). A Chinese environmental NGO representative sums up the connection of the WB to building a domestic CE: “We (China) are doing mandatory waste separation, we have this ban on the import of foreign waste, to promote our own domestic circular industries, all of these, and China also has the Zero-Waste cities concept” (CH_NGO1).

**Table 4:** Popular narrative without institutionalization in China-EU CE cooperation

Narrative	*‘WB as domestic waste management incentive’*	
	Perspectives from China	Perspectives from EU
Problem	China lacks domestic waste collection and sorting systems	EU lacks domestic waste recycling capacities
Cause	Lack of incentives to develop domestic waste collection and sorting system as recyclers import ‘foreign’ waste	Historical dependency on waste exports to China
Consequ-ence	Implementing CE initiatives on waste collection and sorting is challenging	Inability to manage wastes when China stopped waste imports Environmental and reputational damage
Solution	WB cuts off domestic recyclers from foreign supply of scrap	WB cuts off domestic wastes from a major export destination
Benefits	Helps to build Chinese national waste collection and sorting regime Incentivizes waste-importing countries to increase their domestic recycling capacities	Incentivizes waste management capacities within the EU and fosters EU CE Less dependency on China

Agents representing EU perspectives in the ‘*WB as domestic waste management incentive’* narrative stresses that the Waste Ban complements the EU’s CE strategy by cutting off the possibility of shipping wastes to China. It problematizes limited domestic waste recycling capacities within the EU, citing the historical dependency of exporting waste as a cause. These conditions led to the inability to manage wastes when China issued the WB and subsequent environmental and reputational damage. While this narrative acknowledges that in the interim much of the waste has been transferred to other waste-importing countries such as Vietnam and Malaysia, EU CE advocates link the WB to the EU’s Single-Plastic Directive, and argue that the WB gives momentum to the EU’s own CE initiatives, such as increasing domestic waste management capacities (EU_P13, EU_P8, EU_P1). Stakeholders such as this packaging sector representative are convinced that European agents can support the WB: “we could arrange…for that we DON'T have this wastes' exports all over the place, that would be the first thing…certain of this type of practices would simply have to say, ‘This is not—this is not acceptable. This is not—you know, just—just get—let's just get rid of it!’” (EU_I4).

## Discussion

Our discursive analysis of the WB reveals bleak prospects of developing a CE globally or regionally through China-EU cooperation despite China’s successful reconfiguration of global waste and resource politics through strategic narrative shifts. In this section, we provide explanations for why international CE development is currently in a political gridlock.

First, the WB narratives show that China and the EU currently talk past each other in terms of their mutual roles and rules of cooperation. Two divergent WB narratives have opposite perceptions on the disruption caused by the WB and use delegitimating and self-legitimating strategies to gain the upper hand. They show that self and mutual agencies in the waste trade shape their divergent conceptualization of materials in the waste trade shape as well as their opposing perspectives on problems and solutions. China and EU’s conflicting conceptions of mutual agencies are actually founded on an agreement that ‘development is linear’ and ‘trade is not only free of politics but ‘naturally’ conducive to ‘development’. However, though they conceive of their mutual ‘development’ stage similarly – namely that China is slowly ‘catching up’ to ‘developed countries’ – they conceptualize what ‘catch up’ means differently. For the EU, this means that China is no longer a ‘developing country’ (European Commission 2019) and that it should play more by the rules of the ‘developed countries’, comply with global trade norms, and take up more financial responsibility. For China, it sees ‘catch up’ as a transition from being in an underprivileged rule-taker to a more righteous rule-setter and to make up for lost time. In this respect, the ‘development’ narratives are problematic for a global CE as they view industrialization as linear and a hierarchical process (Wade 2016; Rodrik 2018).

The WB is an example of China practicing a key learning from the EU and other developed countries – using environmental standard setting to benefit domestic firms and facilitate structural transformation in its industrial ‘modernization’. This is in line with the ‘leapfrogging’ that scholars have argued the CE has the potential to accomplish for China (Geng and Doberstein 2010; Mathews et al. 2011), although such debates remain contested both in and beyond China. Ultimately, reshaping the global waste regime would require a rethinking of free trade and industrialization as benchmarks for sustainable development, which neither the EU nor China is prepared to do yet. Literature has suggested that globalizing regional environmental policy has been a way to preemptively defend the EU against accusations of protectionism and to ultimately harness market power (Kelemen 2010). In a similar vein, the EU and China’s struggle over the notion of a socio-environmentally fair CE can also be understood as a struggle over economic competitiveness – not necessarily as a mutual commitment towards sustainable policies and practices.

Second, the gridlock is also based on the different prioritization of scales for developing a global waste regime and CE. This gridlock is illustrated as much by the divergent narratives of (de)legitimation as by the inconsistency in EU’s reaction narratives to the WB in general. While China’s WB narrative pushes for a national waste regime in lieu of the global, the EU’s WB narratives is fragmented in its position on its preference of scale. On the one hand, the *‘WB as a destructive disruption’* narrative is closely tied to global ‘free’ trade and thus implies the WB is a barrier to trade and a global CE. On the other hand, agents reproducing this narrative often stress that they are not against the WB but only its implementation, suggesting more allegiance to regionalizing CEs if the EU had more control over its implementation. The popular narrative *‘WB as domestic waste management incentive’* echoes China narratives and builds a coalition with them, using the momentum of the WB to support EU domestic CE efforts. The official China WB narrative, while completely supporting a national CE for wastes, actually pushes for a more globalized supply chain in other sectors such as technology. In sum, the tensions between the WB narratives showcase the weak linkages between a global CE based on global trade and regional or local CEs. This highlights the need for CE scholars and practitioners to conceptualize a global CE that is not reliant on increasing global trade but rather inclusive of regionalized or local circuits of exchange.

Third, the narrative *‘WB as domestic waste management incentive’* refers to how the WB has launched regional CE visions of improved recycling in both China and the EU. While this narrative structures many stakeholders’ arguments and supports China and the EU’s respective domestic waste and CE policies, it is not institutionalized in the international arena and actions that should follow are uncoordinated. Furthermore, this narrative focuses on waste diversion instead of waste prevention, which is problematic because it does not address the extractive socio-environmental problems caused by industrialization and instead propels global waste markets which requires increasing waste volumes to be sustained (O'Neill 2019). End-of-life waste treatment remains a key focus for both narratives. This offers mutual learning opportunities if China and the EU find ways to adapt to their evolving teacher-student relationship, as China and the EU have complementary waste management expertise and experience. Yet, CE scholars have argued that recycling needs to be deprioritized in favour of other strategies such as reuse and repair (Kirchherr et al. 2017; Korhonen et al. 2018). CE policy scholarship on China and the EU have also argued that end-of-life CE policies needs to be complemented with more stringent and consistent policies for input side flows and the entire production life cycle (Zhu et al. 2019; Domenech and Bahn-Walkowiak 2019). Our results provide further evidence for such policy recommendations.

We have discussed three distinct narrative and agency-related explanations for the bleak prospects for coordination between China and the EU. While these explanations are not exhaustive, they provide a nuanced understanding of the political gridlock through a relational lens, going beyond dichotomous presentations of (supra)national interests to examine how interests are constructed and how they can change, thus providing us with possibilities to move forward. This study is limited to addressing WB narratives exclusively in the China-EU CE cooperation within a relatively short time period, it focuses on perceptions and reactions discursively and moves away from focusing solely on statistical trade data. Despite these limitations, our results nevertheless highlight key areas of reflection and negotiation for China-EU relations in the global waste regime and international CE cooperation. We outline them in the conclusion.

## Conclusion

The discursive dynamics found in this study emphasize that it is not only material flows that need to be renegotiated in the global waste regime, but also the very institutions, scales and assessment criteria for a fair and sustainable global CE that addresses capacity disparities between waste-importing and exporting regions. Reshaping this regime would require a rethinking of the role of ‘free’ trade and industrialization as well as their relationship with sustainability, which would mean reconsidering the role of the WTO as a governing institution in this specific policy field. It would also mean rethinking the role of international agreements such as the Basel Convention which focuses on controlling the waste trade for hazardous substances but do not consider what kind of trade is conducive for sustainable development or fail to implement any monitoring capacities. While unilateral national efforts such as Green Fence, National Sword and the WB disrupted the international waste regime, the lack of coordination with other stakeholders within the regime means the socio-environmental outcomes are unpredictable, while environmentally harmful practices are simply shifted onto other places. To achieve more sustainable environmental impact, we argue that new international CE requires new narratives and strategies that reconceptualize the relationship between ‘circularity’ and economic relations across scales, while taking historical discrepancies and the current scenario of urgently needed ecological action into account.

As it may be a long-term process for decision-makers to reimagine their roles beyond a linear development model facilitated by free trade, we argue that, as a first step in the right direction, CE actors need to prioritize circular strategies of waste prevention to ease pressure from the global waste regime as it goes through a necessary transition. Our research highlights three elements that require re-consideration in that process.

First, as China’s self-perceived agency evolves from that of a passive learning ‘student’ to more of an active global environmental leader and ‘teacher’, the EU needs to recognize this shift in order to adapt. Stepping into a new learning role could benefit the building of its own CE – which already has a robust collection and sorting regime but lacks recycling capacities (Qu et al. 2019). Alternatively, a new role could facilitate the EU’s internal renegotiation of its socio-economic metabolism to decrease the waste generated. Such a reevaluation would enable better coordination efforts between the EU and China and give opportunity for discussion of a new waste management regime to emerge that sees individual country’s recycling capacities increased, localizing waste management and shortening distances for scrap circulation. China, for its part, should reevaluate its continued self-perception as a ‘student’ to the EU’s ‘teacher’ with regards to trading environmental technology as a way to reach further stages of industrialization. While the Chinese WB narrative is critical of the EU’s waste exporting practices, it admires the EU’s claim to environmental leadership through green technology exports and globalizing its environmental standard setting. The Chinese WB narrative suggests China’s determination to be the EU’s ‘student’ in this regard leads to international regulatory competition instead of environmental cooperation. Connected to this, a reimagining of the EU and China’s agencies beyond the linear development model is critical because China’s increasing agency questions conventional North–South narratives. Although China’s position in the Global South is debated, its development pathway and achievements are influential for different countries in search of similar outcomes. The ripple effect of the WB on Southeast Asian countries, as many also followed suit in putting up bans, demonstrates this agency.

Second, despite the EU’s focus on the global scale and China’s focus on the national scale, both European and Chinese WB narratives refer to CE visions of waste diversion (internationally and nationally) instead of waste prevention (locally). This focus on the macro scales and related practices of waste burden shifting is problematic because it does not address the extractive socio-environmental problems caused by industrialization and instead propels global waste markets that require increasing waste volumes to be sustained (O'Neill 2019). End-of-life waste treatment remains a key focus for both narratives. While this offers mutual learning opportunities if China and the EU find ways to adapt to the teacher-student relationship, as China and the EU have complementary waste management expertise and experience, it does little for mitigating environmental harm stemming from extraction and use phases of global supply chains.

Third, a reevaluation of agencies would also give opportunity for collaborative redefining of what is ‘waste’ and what is ‘scrap’, enabling trade of some secondary raw materials where necessary but decreasing not only hazardous and illegal waste trades but also working together to find an answer to what materials should be traded at which scale. While the WB’s implementation came as a shock to European countries, renegotiating a multipolar waste regime that is less dependent on any single country, especially China, would be much more stable (Velis 2014). If efforts to repeal the WB fail, the diverted waste from the WB will likely continue to find its way to third countries where there is insufficient capacity to treat the waste in socio-environmentally sustainable ways.

Our analysis has also shown that self and mutual perceptions of agency are important for working relationships in international cooperation towards a CE. China’s unilateral action disrupted the established win–win discourse of global waste trade, showcasing that agents’ self-perception of being a ‘winner’ can change over time, forcing other ‘winners’ to re-evaluate their strategic practices. While the China-EU relationship is crucial for advancing an otherwise fragmented global waste regime, a global CE or different sets of regional CEs would both require the differentiated involvement of many countries in the current global waste regime, not just China and the EU. Further research and practice are needed to investigate and construct narratives pointing to questions such as: What kind of trade (material and financial flows) should occur at which scales (global, regional, local) to achieve sustainable and equitable outcomes in terms of the global waste regime and its contribution to CE(s)? What kind of policy narratives and regulatory frameworks would enable such a transition? How can nation-states co-exist if globalized trade changes in terms of its material qualities, travelling distance, and/or intensity? What kind of regulatory challenges would the WTO face, if such developments were to materialize? Finally, more interlinkages are needed between CE and sustainability research, which highlights issues of recognition, as well as the procedural and distributional aspects of a justice-oriented mode of transitioning (Martin et al. 2020) as being crucial for the reevaluation of waste, trade and environmental governance.

## Supplementary Information

Below is the link to the electronic supplementary material.

Supplementary file1 (PDF 523 KB)
