# Supplementary material for: Explanations of the political gridlock behind international circular economy: Waste Ban narratives in the China-EU cooperation
Source: Ambio. 2022 Jul 19;52(1):126–39. doi: 10.1007/s13280-022-01758-4 (PMC9666588; doi:10.1007/s13280-022-01758-4)
Supplement: Supplementary file 1 — Supplementary file1 (PDF 523 KB) [file 13280_2022_1758_MOESM1_ESM.pdf]

Ambio

Supplementary Information
This supplementary information has not been peer reviewed.

Title:

Explanations for the political gridlock behind international circular economy:

Waste Ban narratives in the China-EU cooperation

Table S1. List of interviewees (anonymized and abbreviated)

Affiliation	Number	Abbreviations
International		
- International Organizations (governmental and non-governmental)	10	IO_1
China		
- Chinese Policy Institutions (Chinese embassies and governmental research institutes)	7	CH_P1
- Chinese Research Institutions	9	CH_R1
- Chinese Industry Associations and Businesses	5	CH_I1
- Chinese Environmental Non-Governmental Organizations	1	CH_NGO1
Europe		
- European Policy Institutions (European Commission, European embassies, Dutch government, regional governments, development agencies and projects)	18	EU_P1
- European Industry Associations and Businesses	15	EU_I1
- European Non-Governmental Organizations	6	EU_NGO1
- European Research Institutions	1	EU_R1
Total	72	
